# Playground Renovations and Quality at Public Parks in Boston, Massachusetts, 1996-2007

**Published:** 2011-06-15

**Authors:** Jessica L. Barrett, Cynthia Hannon, Linda Keefe, Steven L. Gortmaker, Angie L. Cradock

**Affiliations:** Department of Society, Human Development, and Health, Harvard School of Public Health, Landmark Center; University of Massachusetts Boston, Boston, Massachusetts; Fitz Urban Youth Sports, Northeastern University, Boston, Massachusetts; Department of Society, Human Development, and Health, Harvard School of Public Health, Boston, Massachusetts; Department of Society, Human Development, and Health, Harvard School of Public Health, Boston, Massachusetts

## Abstract

**Introduction:**

Recreational and transportation infrastructure can promote physical activity among children and adolescents. The Play Across Boston community-based research project sought to estimate and compare playground renovation rates across Boston areas before and after a playground quality assessment, to describe changes in playground quality among a subset of parks, and to document features of local transportation infrastructure around parks.

**Methods:**

We used an observational pretest-posttest design to estimate playground renovation rates among 103 city-operated parks. Renovation rates were calculated on the basis of annual city Parks Department capital budgets from fiscal years 1996 through 2007. We used the same design to describe changes between a 2000 to 2001 baseline assessment of playground quality and a 2007 follow-up measured via observation of a subsample of 18 low-scoring parks in disadvantaged areas. We used *χ* ^2 ^analysis to compare percentages of playgrounds renovated across city areas before and after baseline assessment, logistic regression analysis to calculate odds ratios comparing renovation rates after baseline by city area, and paired *t* tests to compare playground quality at baseline and follow-up.

**Results:**

Overall playground renovation rates before (29%) and after (34%) baseline assessment were similar. Parks scoring low on playground quality at baseline were renovated after baseline at a higher rate than high-scoring playgrounds. After accounting for baseline playground quality, parks in disadvantaged areas were renovated at a rate similar to those in other areas. Playground quality scores improved between baseline (mean, 38.3; 95% confidence interval, 35.3-41.3) and 2007 in a subsample of previously low-scoring parks in disadvantaged areas.

**Conclusion:**

The findings of the 2007 follow-up assessment indicate an equitable rate of playground renovation across city areas according to need.

## Introduction

Physical activity among children and adolescents can provide lifelong health benefits. Compared with their inactive peers, physically active children and youths have better cardiorespiratory endurance and muscular strength, less body fat, better cardiovascular and metabolic risk factor profiles, stronger bones, and better mental health (1). Physically active youths are more likely to continue a physically active lifestyle into adulthood (2), contributing to reduced risk of several chronic diseases, including obesity, coronary heart disease, hypertension, type 2 diabetes, and some cancers (3).

Features of the built environment can promote physical activity and improve health (4,5). Studies among youths have shown that access to recreational facilities, including parks — in particular, park amenities such as playgrounds — is associated with increased levels of physical activity (6,7). Although playgrounds can provide an inviting setting to promote active play, the quality of playgrounds can be a concern for parents (8) and can vary according to neighborhood socioeconomic characteristics (9).

Transportation infrastructure is also associated with physical activity among youths (5). The presence and condition of sidewalks, presence of controlled street crossings (ie, traffic lights and crosswalks), and existence of road hazards, such as speeding traffic, have all been linked to physical activity levels among youths (5). Traffic engineering countermeasures that reduce the risk of pedestrian and cyclist injuries, such as increasing the visibility of pedestrians and cyclists and reducing vehicle speeds (10), may also promote physical activity. Better traffic control and an infrastructure that promotes pedestrian and bicycle safety have been associated with more frequent walking and cycling to recreational facilities among youths (11), thus creating safe opportunities for them to be physically active, both while using recreational facilities and while traveling to and from them.

Since 1999, the Play Across Boston (PAB) project has worked with community partners to document youth sports and physical activity resources in Boston and to monitor ongoing citywide efforts in reducing documented disparities in access to physical activity facilities and increasing program participation. PAB is a project of the Harvard Prevention Research Center at the Harvard School of Public Health undertaken in collaboration with Northeastern University's Center for the Study of Sport in Society and a broad-based community advisory board. The background of PAB and that of the community process facilitating project development and implementation are described in detail elsewhere (12,13). Briefly, following several community engagement activities, PAB led a comprehensive community-based assessment of physical activity programs and facilities for Boston youths from 1999 through 2001. Between July 2000 and July 2001, PAB staff observed playground quality at 145 public parks in Boston, identified playgrounds most in need of repair or renovation, and found that playground quality varied by socioeconomic and racial composition of local neighborhoods (9).

Beginning in the summer of 2001, assessment findings were disseminated to community leaders to facilitate data-driven action for reducing observed disparities in opportunities for physical activity among youths in disadvantaged neighborhoods (13). In 2007, PAB conducted a follow-up study to describe changes in playground quality after its dissemination of the baseline findings. The objectives of this second study were to 1) estimate and compare playground renovation rates across city areas during the years before and after the PAB baseline playground quality assessment in 2000 to 2001, 2) describe changes in observed playground quality among a subset of parks, and 3) describe features of local bicycle and pedestrian transportation infrastructure that might influence access to public parks.

## Methods

This study used an observational pretest-posttest design with no control arm: 1) to estimate citywide playground renovation rates from fiscal year (FY) 1996 to FY 2007 and 2) to describe changes in playground quality among a subsample of public parks in an area of socioeconomically disadvantaged neighborhoods between a 2000 to 2001 baseline assessment and an August 2007 follow-up. This study also used a cross-sectional design to describe features of local bicycle and pedestrian transportation infrastructure surrounding the parks in the subsample.

### Setting

The setting for this study was Boston, Massachusetts. In the 2000 US Census, Boston had 589,141 residents, 116,559 (20%) of whom were younger than 18 years. Among these youths, 37% were black, 25% white, 24% Hispanic, 7% Asian, and 6% of another race/ethnicity. Twenty-three percent of families with children younger than 18 years had household incomes below the poverty level (14). The percentage of Boston high school students who meet recommended levels of physical activity (30%) is lower than both national (35%) and state (41%) averages and is lower among racial/ethnic minorities than among whites (15).

All 103 public parks operated by the Boston Parks and Recreation Department (Parks Department) that were assessed for playground quality at the PAB baseline were eligible for this study. Forty-two parks that were assessed for playground quality at the PAB baseline were not eligible because they were operated by other city or state agencies.

The city public health agency and community partners have worked with 7 of Boston's 16 neighborhoods to address health promotion around chronic disease risk factors, including physical activity, through the Boston Steps program, funded by the Centers for Disease Control and Prevention, and the Boston Collaborative for Food and Fitness, funded by the W.K. Kellogg Foundation. These 7 neighborhoods are collectively referred to as the "disadvantaged area" in this study. More than two-thirds (69%) of Boston youths live in this disadvantaged area (14). Twice as many residents in this area compared with other city areas are of nonwhite race/ethnicity (64% vs 32%), and a higher percentage of households with children have incomes below the poverty level (25% in the disadvantaged area compared with an average of 19% in other Boston neighborhoods) (14).

Of the 103 eligible parks, 59 were in the disadvantaged area. For follow-up assessment of playground quality we selected all parks located in the disadvantaged area that had a baseline playground quality score lower than 50 (n = 19). We considered playgrounds scoring below 50 (n = 24 citywide) to be low-scoring playgrounds in need of repairs or renovations. Of the 19 parks selected for the subsample, 1 park was not assessed in 2007 because of ongoing renovations, resulting in a subsample total of 18 parks for analysis.

### Measures

We reviewed Parks Department capital budgets from FY 1996 through FY 2007 to estimate citywide playground renovation rates among the park sample. We obtained annual capital budgets from the city of Boston website (www.cityofboston.gov/budget/) and the Boston Public Library Government Documents Department. The Parks Department schedules repair and replacement of parks on the basis of these annual capital budgets. We reviewed projects listed in the capital budgets and identified projects as playground renovations if 1) the project description included the term “playground,” “playlot,” “totlot,” or “play equipment” and 2) the anticipated completion date listed was in that budget year (for FY 1996-FY 1998 budgets) or the project status was listed as “completed” or “in construction” (for FY 1999-FY 2007 budgets). Different criteria were required for budgets in FY 1996 through FY 1998 and FY 1999 through FY 2007 because the budget format changed.

After identifying playground renovations, we calculated renovation rates for the 6-year periods before (FY 1996-FY 2001) and after (FY 2002-FY 2007) the PAB baseline assessment. The renovation rate in each period equaled the total number of parks renovated in the period divided by the total number of parks in the sample. We used 6-year rates instead of annual rates because some renovation projects spanned multiple budget years.

Researchers conducted a follow-up assessment of parks to assess improvement in playground quality from baseline to follow-up. A research assistant (J.L.B.) visited a subsample of parks during August 2007 and assessed playground quality by using the same playground quality instrument used in the baseline assessment. This instrument (9,12) includes 24 items rating climbing equipment, swings, sandboxes, spray pools, and ease of supervision of children in play areas according to safety standards of the city of Boston (16), the US Public Interest Research Group (17), and the US Consumer Product Safety Commission (18). The instrument has previously demonstrated good inter-rater reliability (*r* = 0.77) and 4-month test-retest reliability (*r* = 0.71) (9). Researchers calculated a playground quality score for each park to indicate the percentage of playground features meeting the quality standards. Higher scores indicate better playground quality.

During the assessment of playground quality in August 2007, the same research assistant also assessed bicycle and pedestrian access around the subsample of parks. The purpose of this assessment was to pilot-test a tool for assessing infrastructure features around parks and to investigate features potentially influencing access to public parks. Using an instrument adapted from existing tools (19-21), we observed features of streets, sidewalks, intersections, and marked crosswalks on street blocks containing park entrances. Community partners in city parks departments, public health agencies, and pedestrian advocacy organizations informed development of this instrument. We averaged compliance with each item assessed in each park to assign parks a score from 0 to 100 for that item. For example, a score of 50 for good sidewalk condition might indicate that 2 of 4 sidewalk lengths assessed were in good condition. Researchers calculated a total bicycle and pedestrian access score for each park to indicate the percentage of items assessed that met recommendations for promoting safe, active park access. Higher scores indicate better local environments.

### Analysis

We used the McNemar test to compare overall renovation rates before and after baseline among the parks sampled and *χ* ^2^ analysis for comparison by city area and by playground quality score for those parks not renovated at baseline. Among the latter, we used logistic regression analysis to calculate odds ratios to compare renovation rates after baseline by city area, controlling for baseline playground quality score. All analyses were conducted with SAS version 9.1 (SAS Institute, Inc, Cary, North Carolina). Significance was set at *P* < .05.

We performed a new analysis of baseline playground quality scores to calculate the mean score in the follow-up park sample and to compare scores by city area using a *t* test. We performed a paired *t* test to compare the mean difference in scores between baseline and follow-up of parks in the subsample that were assessed for playground quality at follow-up. We calculated the percentage of parks meeting standards for each bicycle and pedestrian access item and the mean total score among the subsample of parks.

## Results

### Playground renovation rates

Parks Department capital budgets documented that 62 of the 103 Parks Department-operated playgrounds assessed in the PAB baseline assessment were renovated in the 12-year period from FY 1996 through FY 2007 (Table 1). Three playgrounds were renovated both before and after PAB baseline. Citywide, playground renovation rates before (29%) and after (34%) PAB baseline assessment and dissemination activities were similar (P = .34). Compared with parks in other city areas, parks in the disadvantaged area tended to be renovated at a slightly lower rate before baseline. After baseline, 39% of parks in the disadvantaged area were renovated, compared with 27% in other city areas (P = .21). Among the 73 parks not renovated before baseline, parks scoring low at baseline were renovated after baseline at a higher rate compared with parks scoring higher at baseline (odds ratio, 8.16;95% CI, 2.55-26.16; P < .001). Controlling for baseline score, parks in the disadvantaged area that were not renovated before baseline were renovated after baseline at a rate similar to that of other city areas (P = .97).

### Playground quality

Parks in the disadvantaged area had lower overall baseline playground quality scores compared with parks in other city areas (*P* < .001) (Table 2). In the subsample of parks assessed at follow-up, average playground quality scores improved between the baseline (38.3) and follow-up (64.6) assessments (mean difference, 26.3; 95% CI, 17.8-34.7; P < .001). At follow-up, most of these playgrounds were constructed with safety features to prevent injury from falling, tripping, and entrapment and to allow for adult supervision of children using equipment. Some playground quality measures, such as appropriate safety surfacing and lack of debris under equipment, broken or missing parts, or peeling or chipping paint, continued to have low compliance with the standards assessed. According to Parks Department capital budgets, 2 of the 18 parks were renovated from FY 1996 to FY 2001, 13 from FY 2002 to FY 2007, and 3 were not renovated during this period.

### Bicycle and pedestrian access

Bicycle and pedestrian access scores suggest that, on average, these parks met standards for approximately half of the items assessed (mean, 52.3; 95% confidence interval, 47.7-56.9) (Table 3). Parks frequently had well-lit entrances and were surrounded by sidewalks that were sufficiently wide. Marked crosswalks on street blocks containing park entrances were often visible, and pedestrian signals at marked crosswalks always provided adequate crossing time. On the other hand, neither bicycle racks nor bicycle lanes (ie, special lanes marked on the street for cyclists) were present on any street blocks containing park entrances. Only 29% of street blocks assessed had speed limits below 30 mph. Intersections defining blocks containing park entrances rarely employed traffic-calming measures such as speed humps, curb extensions, or other engineering features designed to slow oncoming traffic and improve pedestrian safety. Few of the available marked crosswalks were marked with pedestrian-related signage to identify pedestrian crossing locations for drivers, and no sidewalks leading into marked crosswalks had detectable warnings underfoot to alert pedestrians to the crossing transition.

## Discussion

In previous findings from the 2000 to 2001 baseline assessment, neighborhoods with lower playground quality scores also tended to have fewer playgrounds in proportion to youth population (9), suggesting that playgrounds in these neighborhoods may experience more wear and require more frequent maintenance. Between 2000 to 2001 and 2007, PAB staff met with Boston residents and with the Boston mayor's office and the Parks Department to share a complete list of observed playground quality scores, and released a report summarizing key findings (12,13). The results of our follow-up assessment suggest that the Parks Department renovation schedule has equitably addressed playgrounds according to need and that the quality of playgrounds studied improved between 2000 to 2001 and 2007. In this study, we observed lower playground quality at baseline among city playgrounds in a disadvantaged area compared with other city areas. Our finding that before baseline playground assessment, playgrounds in the disadvantaged area were renovated at a rate similar to that of other city areas, suggests that differences in baseline playground quality across areas may be due to differences in use and maintenance rather than construction. We found that among parks that had not been renovated before baseline, those identified as in need of renovation based on low playground quality scores were renovated at a significantly higher rate than those with higher quality scores. Furthermore, accounting for baseline quality, playgrounds were replaced according to need in both disadvantaged and other city areas after the initial playground assessment.

Results from the follow-up observational assessment corroborate findings regarding playground renovations. Many of the playground quality items that improved between the baseline and follow-up assessments were related to playground equipment construction, suggesting that newer installations are meeting more stringent safety standards. The playground quality items that continued to have low compliance were largely related to maintenance issues, such as upkeep of safety surfacing and attention to broken or missing equipment.

Findings regarding bicycle and pedestrian access offer a starting point for further dialogue and collaboration to provide and improve local bicycle and pedestrian access to these playgrounds and parks, which are destinations for children and adults alike. Overall, pedestrian access appeared better than bicycle access around the parks assessed. Road lighting and sidewalks, basic infrastructure associated with reductions in pedestrian injuries (10), were found surrounding nearly all parks. However, other features that can increase pedestrian safety, such as traffic calming measures (22), could be improved. Cheaper and simpler strategies, such as installing pedestrian-related signage to identify pedestrian crossings for drivers, are feasible first steps toward improving the infrastructure. The addition of bicycle racks in and around parks will make cycling to these destinations more attractive. In 2007, the Boston Mayor's Office began several citywide initiatives promoting bicycling and has added 1,500 bicycle parking spaces and 33 miles of bicycle lanes throughout Boston neighborhoods since our assessment (23).

Several limitations to this study should be noted. The study was conducted in 1 city by using an observational design without a comparison community. Therefore, we cannot account for natural history, particularly with respect to the overall economic climate and the lifespan of playgrounds. We did not know the age of each playground at baseline, which would likely influence observed playground quality and renovation scheduling, because we lacked detailed capital budget data before FY 1996. Analyses of playground renovations after baseline were limited by small sample size and produced wide confidence intervals; point estimates should be interpreted with caution. The measure used to determine playground renovation rates was not assessed for reliability or validity. We lacked detailed project descriptions and did not confirm project construction dates through discussion with city officials or through direct observation, except among the subsample of parks observed. We also did not examine other funding sources for playground renovations, which could vary by park location. Future studies could compare playground renovation history across communities, validate the method for identifying playground renovations by using city Parks Department capital budgets, and investigate other sources of park renovation funding.

For observational assessments, results are representative only of playgrounds in the disadvantaged area that had low observed playground quality at baseline. Because of resource limitations, we were not able to observe playground quality or bicycle and pedestrian access citywide or make comparisons by city area or baseline quality with sufficient power. A comprehensive assessment of all Boston playgrounds is needed to describe citywide changes in playground quality. In the playground quality instrument used, items contribute equally to the playground score, so the same score for 2 different parks or for the same park at baseline and follow-up may not reflect equivalent injury hazard. At follow-up, 1 researcher (J.L.B.) completed all playground quality assessments, whereas at baseline 5 researchers (including C.H. and A.L.C.) assessed quality. However, 1 researcher (A.L.C.) assessed 64% of parks at baseline, and the instrument demonstrated good interrater reliability (*r* = 0.77) (9). The bicycle and pedestrian access instrument was not tested for reliability or validity. Also, bicycle and pedestrian access scores do not account for parent or child perceptions of the environment, potentially significant factors affecting decisions about active transportation (5). Future studies could assess validity of the bicycle and pedestrian access instrument and investigate transportation infrastructure on a larger scale and across a larger geographic area.

One of many ways to promote physical activity and health among children and youths is through provision of accessible, quality recreational infrastructure, including playgrounds. In this study, data from Parks Department capital budgets indicate an equitable rate of playground renovation across city areas according to need, as observed and communicated through the PAB community-based research project.

## Figures and Tables

**Table 1:** Rates of Playground Renovations Among City of Boston Parks Included in Baseline Playground Assessment (N = 103) in 2000 to 2001^a^

Park Category	No. of Parks	Renovations During 1996-2001, Before Baseline Assessment		Renovations During 2002-2007, After Baseline Assessment	

		No. of Playgrounds Renovated	Renovation Rate,^b^^ %^	No. of Playgrounds Renovated	Renovation Rate,^b^^ %^
**Renovated during 1996-2001 or during 2002-2007**					
Total^c^	103	30	29	35	34
**By city area**					
Parks in disadvantaged areas^d^	59	15	25	23	39
Other parks	44	15	34	12	27
**Renovations only during 2002-2007**					
Total	73	0	0	32	44
**By baseline playground quality score^e^**					
Low score (<50%)	22	0	0	17	77
High score (≥50%)	51	0	0	15	29
**By city area**					
Parks in disadvantaged areas^d^	44	0	0	21	48
Other parks	29	0	0	11	38

a Source of all data is Boston Parks and Recreation Department capital budgets, fiscal year 1996 through fiscal year 2007. A baseline playground assessment was performed by Play Across Boston in 2000 to 2001; not all city parks included in the assessment were included in this study.

b The playground renovation rate is the number of parks renovated at any point during 1996-2001 or during 2002-2007 divided by the total number of parks.

c Total playgrounds renovated during 1996-2001 and during 2002-2007 do not sum to total playgrounds renovated during 1996-2007 (n = 62) because 3 parks were renovated during both time periods.

d Disadvantaged areas were 7 neighborhoods targeted by the city public health agency. Other city areas were all other neighborhoods.

e The baseline playground quality score is the proportion of items that complied with safety standards for climbing equipment, swings, sandboxes, and spray pools and ease of supervision of children in play areas.

**Table 2:** Mean Baseline Scores for Play Across Boston Playground Quality Assessment, 2000 to 2001^a^

**Park Characteristic**	n	Mean % (95% CI)
**Overall (N = 103)**		60.7 (57.4-64.1)
**In disadvantaged area^b^**		
Overall	59	55.5 (51.4-59.6)
Low baseline score (<50%)^c^	19	38.4 (35.5-41.2)
High baseline score (≥50%)	40	63.6 (59.9-67.4)
**In other city areas^d^**		
Overall	44	67.8 (62.6-72.9)
Low baseline score (<50%)^c^	5	35.9 (23.6-48.2)
High baseline score (≥50)	39	71.8 (67.6-76.0)

Abbreviation: CI, confidence interval.

a Playground quality score indicates ease of supervision of children in play areas and proportion of playground quality items assessed that complied with safety standards for climbing equipment, swings, sandboxes, and spray pools.

b Defined as 7 neighborhoods targeted by the city public health agency and community partners for health promotion around chronic disease risk factors, including physical activity.

c The baseline playground quality score is the proportion of playground quality items assessed that complied with safety standards for climbing equipment, swings, sandboxes, and spray pools and ease of supervision of children in play areas.

d Defined as all city neighborhoods not in disadvantaged area.

**Table 3:** Percentage of Parks Meeting Standards for Bicycle and Pedestrian Access Characteristics Assessed on Street Blocks With Park Entrances, Play Across Boston, 2007

**Bicycle/Pedestrian Access Characteristic**	**Description**	No. of Parks Assessed for Characteristic	% of Parks Meeting Standard for Characteristic
Bicycle racks	Bicycle rack present in or around park	18	0
Bicycle lane	Bicycle lane (ie, pavement marking designating a street lane for cyclists) present on street segments around park	18	0
Slow speed limit	Speed limit on street segments less than 30 mph	18	29
Sidewalk present	Sidewalk present on street segments	18	100
Good sidewalk condition	Sidewalk condition coded as "smooth" or "some small bumps/cracks"	18	75
Recommended sidewalk width	Sidewalk on street segments at least 5 feet wide	18	96
Lighted entrance	Lighting present within 40 feet of entrance	18	90
No cars parked near entrance	Entrances free of nearby parked cars, which might limit visibility for vehicles and pedestrians	18	62
Crosswalk presence near entrance	Crosswalk present within 1 block of entrances, among entrances leading to the street	18	48
Vehicle traffic control	Vehicle traffic control (ie, stop signs and traffic lights) present at intersections around parks	18	46
Few street lanes	No more than 2 travel lanes on the main street at intersections around parks	18	96
Traffic calming	Traffic calming (eg, bulbouts, roundabouts, raised intersections or crosswalks, landscaping) employed at intersections	18	3
Crosswalk at controlled intersections^a^	Crosswalks present at intersections with vehicle traffic control	15	51
Visible crosswalks^b^	Crosswalks visible (coded as "highly visible" or "sufficiently visible")	12	87
Push-button pedestrian signals^c^	Push-button-activated pedestrian signal present at crosswalks at intersections with vehicle traffic control, alerting pedestrians to appropriate crossing time	9	52
Recommended crossing speed^d^	Pedestrian signal allowing crossing speed of less than 4 feet per second	7	100
Pedestrian-related signage^b^	Signage present at crosswalks to alert drivers to pedestrian crossing locations (ie, "Pedestrian Crossing")	12	21
Curb ramps^b^	Curb ramps with a sloped transition to lead pedestrians from sidewalk to crosswalk	12	81
Detectable warnings at curb ramps^b^	Truncated domes present on curb ramps to alert pedestrians to the transition from sidewalk to crosswalk	12	0

a 3 parks were located on street blocks containing no controlled intersections.

b 6 parks were located on street blocks containing no crosswalks.

c 9 parks were located on street blocks containing no crosswalks at controlled intersections.

d Of the 9 parks assessed for presence of push-button pedestrian signals, 2 contained none.
